# Effects of Water and Nitrogen Addition on Species Turnover in Temperate Grasslands in Northern China

**DOI:** 10.1371/journal.pone.0039762

**Published:** 2012-06-29

**Authors:** Zhuwen Xu, Shiqiang Wan, Haiyan Ren, Xingguo Han, Mai-He Li, Weixin Cheng, Yong Jiang

**Affiliations:** 1 State Key Laboratory of Forest and Soil Ecology, Institute of Applied Ecology, Chinese Academy of Sciences, Shenyang, China; 2 Key Laboratory of Plant Stress Biology, College of Life Sciences, Henan University, Kaifeng, China; 3 State Key Laboratory of Vegetation and Environmental Change, Institute of Botany, Chinese Academy of Sciences, Beijing, China; 4 Forest dynamics, Swiss Federal Research Institute WSL, Birmensdorf, Switzerland; 5 Environmental Studies Department, University of California Santa Cruz, Santa Cruz, California, United States of America; Lakehead University, Canada

## Abstract

Global nitrogen (N) deposition and climate change have been identified as two of the most important causes of current plant diversity loss. However, temporal patterns of species turnover underlying diversity changes in response to changing precipitation regimes and atmospheric N deposition have received inadequate attention. We carried out a manipulation experiment in a steppe and an old-field in North China from 2005 to 2009, to test the hypothesis that water addition enhances plant species richness through increase in the rate of species gain and decrease in the rate of species loss, while N addition has opposite effects on species changes. Our results showed that water addition increased the rate of species gain in both the steppe and the old field but decreased the rates of species loss and turnover in the old field. In contrast, N addition increased the rates of species loss and turnover in the steppe but decreased the rate of species gain in the old field. The rate of species change was greater in the old field than in the steppe. Water interacted with N to affect species richness and species turnover, indicating that the impacts of N on semi-arid grasslands were largely mediated by water availability. The temporal stability of communities was negatively correlated with rates of species loss and turnover, suggesting that water addition might enhance, but N addition would reduce the compositional stability of grasslands. Experimental results support our initial hypothesis and demonstrate that water and N availabilities differed in the effects on rate of species change in the temperate grasslands, and these effects also depend on grassland types and/or land-use history. Species gain and loss together contribute to the dynamic change of species richness in semi-arid grasslands under future climate change.

## Introduction

Species turnover directly determines changes in species richness across time, and reflects the dynamic stability of a community [1]–[4]. Effects of changed precipitation regimes and increased atmospheric nitrogen (N) deposition on species composition and diversity of plant communities are crucially important and have increasingly been studied [5]–[11]. However, the temporal patterns of species turnover leading to changes in species composition and diversity are less understood. Species turnover has been found to be strongly dependent upon water [12] and nutrient status [13], [14] in grasslands. It has been previously predicted that the rate of changes for dominant species and life forms is faster on nutrient-rich sites than on nutrient-poor sites [15]–[17]. Nutrient enrichment tends to delay species replacement during a succession from annual to perennial herbaceous species [18], [19]. Most of such findings are based on theoretical models [2], [12] or investigations using natural experiments without environmental manipulation [3], [15]–[17]. Direct manipulative experimental evidences for effects of water and N availability on species turnover are still lacking.

Temperate grasslands in northern China are suitable sites for manipulative experiments since the summer precipitation [20] and the atmospheric N deposition [21] are predicted to increase in the coming decades. These grasslands support diverse species of plants and animals and play important roles in servicing the ecological environment and socio-economics of the region [22]. These grassland ecosystems are sensitive to N enrichment and increased precipitation in terms of plant traits, community structure, species composition, biodiversity, and ecosystem functioning [11], [23]–[26]. To our knowledge, however, no studies have experimentally investigated the effects of water and N addition and their interaction on plant species turnover in these grasslands. Hence, we carried out an experiment with N and water manipulations in two typical types (a steppe and an old field) of that temperate grassland from 2005 to 2009, to explore how water and N enrichments and their interaction affect plant species gain, loss, turnover, and the rate of species change. Given positive effects of water addition on species richness [6], [7], [9], [27], [28] and negative effects of N addition on diversity [5], [8], [29], [30], we hypothesized that water addition enhances plant species richness through increase in the rate of species gain and decrease in the rate of species loss, while N addition has opposite effects on species changes in the temperate grasslands. This study will provide direct experimental evidence for effects of water and N availabilities on dynamic change in species richness, and contribute to predicting the effects of global changes on grassland ecosystems across scales.

## Methods

### Study Sites and Experimental Design

The study sites were located near the Restoration Ecological Research Station (116°17′ E and 42°02′ N, elevation 1324 m a.s.l.) of Institute of Botany, Chinese Academy of Sciences (IBCAS), in Duolun county, Inner Mongolia. Mean annual precipitation is 379.4 mm and mean annual temperature is 2.1°C, with mean monthly temperature ranging from −17.5°C in January to 18.9°C in July. Soil is classified as Calcis-orthic Aridisol according to the US Soil Taxonomy classification. All necessary permits for the described field study have been obtained from the IBCAS at the beginning of the experiment.

Over-grazing and intensive farming in the region for the last 50 years have resulted in severe land degradation and desertification [31], [32]. Within that area, two existed typical types of grasslands, i.e. a steppe and an adjacent old field, were selected for the present study. Both grassland systems were similarly grazed before the old field was converted to farmland in early 1980 s. *Sesamum indicum* L., *Avena chinensis* (Fisch. ex Roem. et Schult.) Metzg., *Triticum aestivum* L., and *Fagopyrum sagittatum* Gilib. were common crops in the old field until 2000. The steppe was overgrazed and severely degraded until it was fenced in 2000, and the old field was abandoned and fenced in the same year, when the local government started to protect the environment from over-grazing and further degradation [33]. Both grasslands have not been used in any form since 2000. At the beginning of the present experiment, the dominant plant species were *Agropyron cristatum* (L.) Gaertn., followed by *Artemisia scoparia* Waldst. et Kit. in the old field, and *Artemisia frigida* Willd., *A. cristatum* (L.) Gaertn., and *Stipa krylovii* Roshev. in the steppe.

A split-plot experimental design was employed in this study. Seven 107 m×8 m blocks were set up within each of the two homogeneous grasslands (30 ha for the steppe, and 15 ha for the old field) in 2005. Each block was divided into two main plots with water treatment (ambient precipitation and water addition). Each main plot was divided into six subplots. Nitrogen treatment (N addition vs. control without N addition) was randomly assigned to each subplot within each main plot. One meter buffer zone between any two subplots was remained. In the middle growing season from June to August, the water addition plots received 15 mm of precipitation weekly by sprinkling irrigation. A total of 180 mm precipitation, approximately 50% of mean annual rainfall, was added yearly during the growing season from 2005 to 2009. Each subplot treated with N addition received 10 g N m^−2^ yr^−1^ in the form of urea, half of which was applied in early May and the other half was applied in late June from 2005 to 2009.

### Plant Community and Litter Biomass Measurements

In May 2005, a permanent quadrat of 1 m×1 m was established in each subplot. Plant survey was conducted consistently within each quadrat in mid-July from 2005 to 2009, to record the plant species and to determine species richness. Percentage of plant-covered and bare ground was measured in each quadrat using a 1 m×1 m metal pane with 100 equal grids (10 cm×10 cm each), by counting the grid junctions whose vertical projections overlapped with plant species or bare ground. Plant coverage was visually carefully estimated for species that did not present at the junctions or presented at the junctions but occupied only very small area in the quadrat. To identify the functional group composition of plant community, species were classified into grasses and forbs according to their life forms. We also counted the annuals and biennials (AB) and perennials for documenting species replacement during the experiment period of 5 years. In early September from 2007 to 2009, plant litter accumulation was collected within a 2 m×0.15 m quadrat in each subplot, and dry litter mass was determined after oven-drying to a constant weight. To describe the rate of changes in species composition, we calculated percentage species gain rate (*G*_p_), loss rate (*L*_p_) and turnover rate (*T*_p_), according to Anderson [2]:
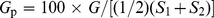


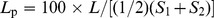


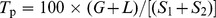
where *G* and *L* are the number of new species gained and old species lost between any two investigation dates, respectively, and *S*_1_ and *S*_2_ are the total species number recorded in the beginning and the end of that investigation, respectively. We calculated interannual *G*_p_, *L*_p_, and *T*_p_, using species data recorded from 2005 to 2009. Because of the frequent instances of no net changes in species gained or lost in interannual data as reported in a study in boreal forest in Australia [34], we also presented the species change rate across 5 years, using data collected in 2005 and 2009. A species that disappeared and later reappeared was excluded in *G*_p_, *L*_p_ and *T*_p_ calculations [2].

### Statistical Analysis

To determine the plant coverage of each functional group (grass and forb) for each quadrat, plant coverage values for species belonging to the same functional group were summed. Total community cover was calculated as 100 minus value of the bare ground cover within each subplot. Temporal stability of a community was computed as mean total community cover across the study period of five years divided by its standard deviation [35].

Repeated measures ANOVAs with a split-plot design were performed to test the effects of block, water, N, year, and their interactions on species richness, litter biomass, and cover of grasses and forbs. Between-subject effects were evaluated as block, water, N, and their interactions, and within-subject effects were year and its interactions with water and N. Univariate process of General Linear Model with a split-plot design was executed to determine the main effects of block, water and N addition and their interactions on rates of changes in species number, and numbers of grasses and forbs gained and lost. The statistical significance of the observed divergence between the steppe and the old field and between grasses and forbs was investigated by the *t*-test at the 5% level. Simple linear regression analysis was used to determine the contribution of individual species cover to variations in the cover of functional group in each grassland, and to examine the relationships between rates of changes in species number and the temporal community stability. Regression statistics used square root transformed data to meet the assumptions of normality and homogeneity. All statistical analyses were conducted using SPSS 13.0 (SPSS, Inc., Chicago, IL, U.S.A.).

## Results

### Changes in Species Richness

**Figure 1:**
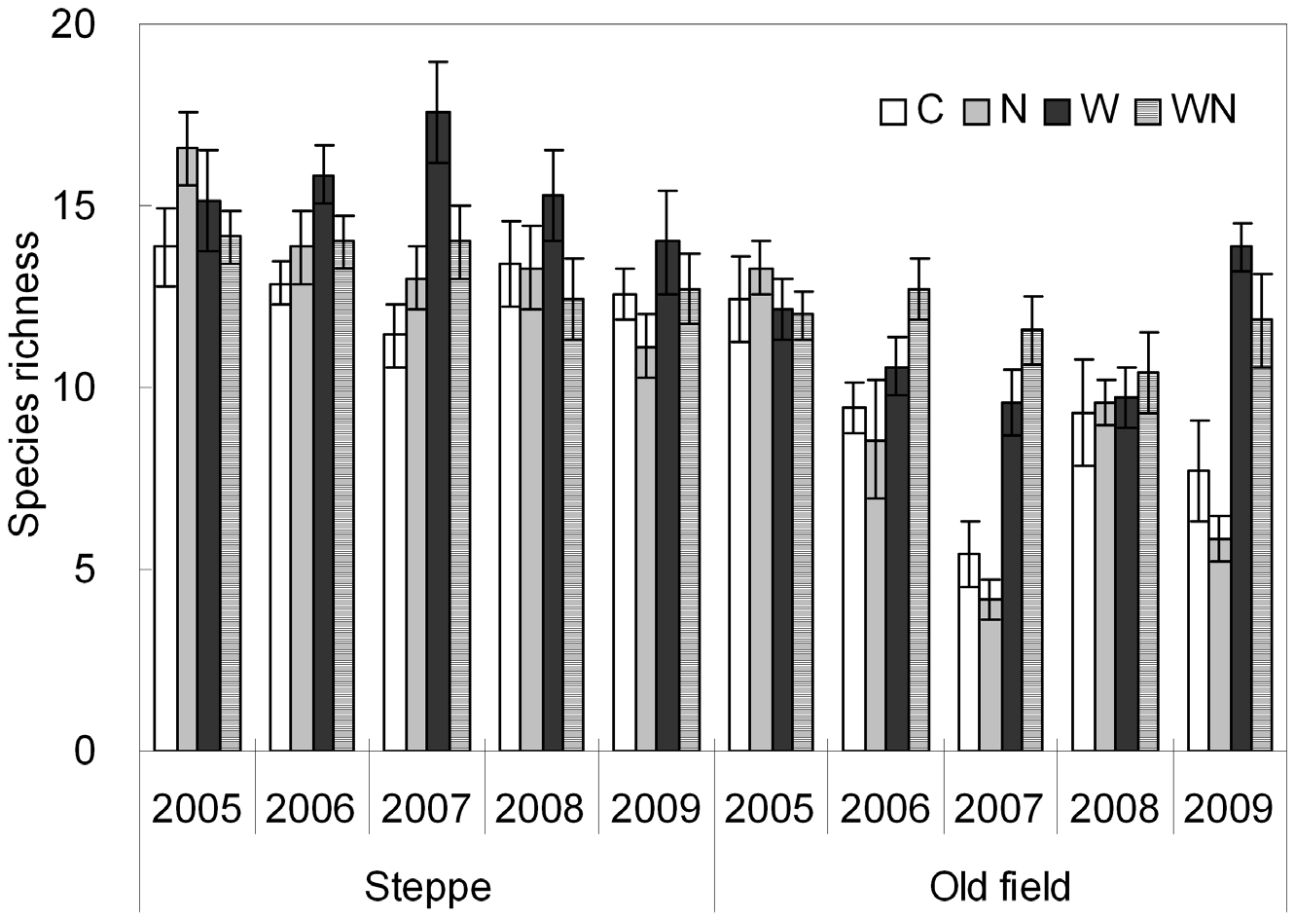
Species richness (mean ± SE) in relation to water and nitrogen addition in a steppe and an old field from 2005 to 2009. C: control, N: nitrogen addition, W: water addition, WN: combination of water and nitrogen addition.

There were significant interannual variations in species richness for both the steppe and the old field site (both *P*<0.001) during the study period (Table 1). Across the treatments, the mean species richness decreased from 14.9 to 12.6 in the steppe, and from 12.5 to 9.8 (with the smallest richness value of 7.7 in 2007) in the old field from 2005 to 2009 (Fig. S1A). Species richness was significantly higher in the steppe than in the old field (*t*-test, *P*<0.001; Fig. 1, S1A,B). During the study period of five years, water addition increased species richness from 13.2 to 14.5 (*P*<0.1) in the steppe and from 8.6 to 11.4 (*P*<0.05) in the old field (Table 1; Fig. S1B). N addition did not affect species richness in both grasslands (Table 1; Fig. 1A). The effect of water addition on species richness was marginally significant (*P*<0.1) depending on N treatments in the steppe but independent of N treatments in the old field (Table 1). The species richness of annuals and biennials (AB) in the steppe was much less than that in the old field (*t*-test, *P*<0.001; Fig. S2A). In both the steppe and the old field, AB richness showed a significant decline (*P*<0.001) with time during the experimental period except for 2007 (Fig. S2A). The extremely dry year of 2007 resulted in a marked decrease in AB species richness. For the perennials, a weak trend of increase in richness existed in the old field, but no consistent pattern was found in the steppe (Fig. S2B).

**Table 1:** Results (*F*-value) of repeated measures ANOVA with a split-plot design on the effects of block (B), year (Y), water (W) and N addition on species richness, litter biomass and plant cover of grasses and forbs in a steppe and in an old field grassland studied from 2005 to 2009.

Source	Species richness		Litter biomass		Grasses cover		Forbs cover		
	Steppe	Old field	Steppe	Old field	Steppe	Old field	Steppe	Old field	
B	1.97ns	0.87ns	2.12ns	2.99ns	1.56ns	8.62*	0.69ns	9.31*	
W	3.56Λ	11.48*	2.15ns	6.77*	0.53ns	11.24*	35.71***	23.73**	
N	0.97ns	0.00ns	12.53*	3.78Λ	34.73**	26.07**	3.53 ns	2.2ns	
W×N	4.21Λ	0.43ns	0.61ns	5.46ns	0.08ns	0.05ns	8.08*	2.5ns	
Y	7.43***	18.98***	2.06ns	12.71**	19.6***	40.27***	27.67***	18.04***	
Y×W	6.08**	15.11***	6.54*	4.50*	6.7***	8.29***	3.3Λ	2.87*	
Y×N	2.36ns	1.89ns	1.23ns	0.55ns	1.75ns	3.78*	4.92*	0.79ns	
Y×W×N	2.42ns	1.50ns	1.62ns	1.08ns	1.05ns	0.73ns	0.93ns	0.54ns	

* , **, *** indicate statistically significant difference at *P*<0.05, 0.01, and 0.001, respectively;

Λ marginally significant difference at *P*<0.1;

ns: *P*>0.1.

### Variation in Rate and Number of Species Change

**Figure 2:**
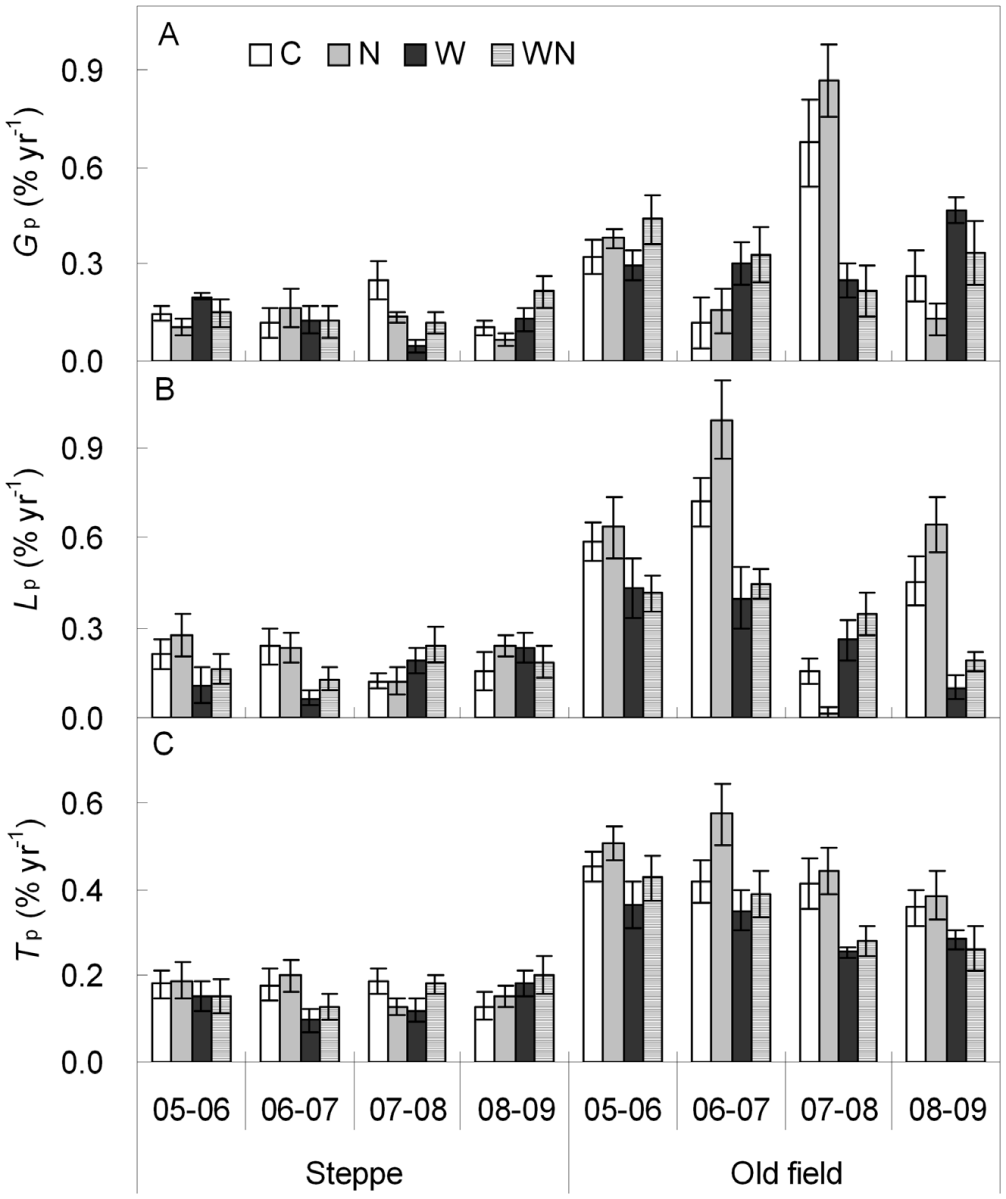
Responses of interannual rate of species change (*G*_p_  =  gain rate, *L*_p_  =  loss rate, *T*_p_  =  turnover rate) (mean ± SE) to water and nitrogen addition in a steppe and an old field from 2005 to 2009. See Fig. 1 for treatment abbreviations.

Inter-annual changes in the rates of species gain and loss (*G*_p_ and *L*_p_) were affected by water and N addition for both grasslands (Table 2). Water addition significantly influenced inter-annual *G*_p_ and *L*_p_ in the steppe (except for 06–07 *G*_p_ and 08–09 *L*_p_) and in the old field (except for 05–06 *G*_p_) (Table 2). Compared to water availability, N affected only 05–06 G_p_ (*P*<0.05) and 05–06 *L*_p_ (*P*<0.1) in the steppe, and 05–06 *G*_p_ (*P*<0.1), 08–09 *G*_p_ and 08–09 *L*_p_ (both *P*<0.01) in the old field (Table 2). Water addition did not affect *T*_p_ at a *P*<0.05 level in the steppe, but significantly (*P*<0.05) influenced *T*_p_ for 07–08 (*P*<0.01) and 08–09 (*P*<0.05) in the old field (Table 2). N addition had no effect on *T*_p_ in both grasslands (Table 2). However, overall trend showed that the rate of interannual changes in species was very small, with the maximal rate of interannual *G*_p_ of <0.9% yr^−1^, *L*_p_ of <1.0% yr^−1^, and *T*_p_ of <0.6% yr^−1^ (Fig. 2). Interannual *G*_p_, *L*_p_ and *T*_p_ were consistently greater (*P*<0.05) in the old field than in the steppe expect for *L*_p_ for 2007–2008 (*t*-test, *P*>0.1).

**Table 2:** Results (*F*-value) of three-way ANOVAs with a split-plot design on the effects of block (B), water (W) and N addition on interannual species change rate (*G*_p_  =  gain rate; *L*_p_  =  loss rate; *T*_p_  =  turnover rate) in a steppe and in an old field studied from 2005 to 2009.

		Steppe				Old field			
		05–06	06–07	07–08	08–09	05–06	06–07	07–08	08–09
*G_p_*	B	6.90*	1.45ns	0.36ns	1.37ns	1.39ns	1.23ns	2.57ns	0.75ns
	W	10.88*	0.13ns	14.22**	6.41*	0.25ns	4.40Λ	39.64***	36.56***
	N	10.52*	0.13ns	0.96ns	0.56ns	4.81Λ	0.17ns	0.84ns	15.68**
	W×N	0.05ns	0.87ns	10.21*	3.13ns	1.16ns	0.01ns	1.81ns	0.00ns
*L_p_*	B	6.70*	0.60ns	2.06ns	1.02ns	3.65Λ	1.85ns	0.60ns	6.79*
	W	17.22**	9.60*	7.82*	0.06ns	5.68Λ	16.48**	17.45**	129.99***
	N	4.56Λ	0.80ns	0.03ns	0.11ns	0.00ns	1.92ns	0.20ns	14.94**
	W×N	0.70ns	0.62ns	0.56ns	1.53ns	0.08ns	0.85ns	4.82Λ	1.93ns
*T_p_*	B	12.19**	0.92ns	1.13ns	0.56ns	4.35Λ	0.31ns	3.90Λ	2.40ns
	W	3.13ns	4.43Λ	0.18ns	1.56ns	4.21Λ	2.05ns	24.08**	11.26*
	N	0.08ns	0.82ns	0.00ns	0.29ns	1.88ns	1.11ns	0.76ns	0.00ns
	W×N	0.07ns	0.00ns	6.30*	0.01ns	0.13ns	0.38ns	0.01ns	0.70ns

* , **, *** indicate statistically significant difference at *P*<0.05, 0.01 and 0.001, respectively;

Λ marginally significant difference at *P*<0.1;

ns: *P*>0.1.

**Figure 3:**
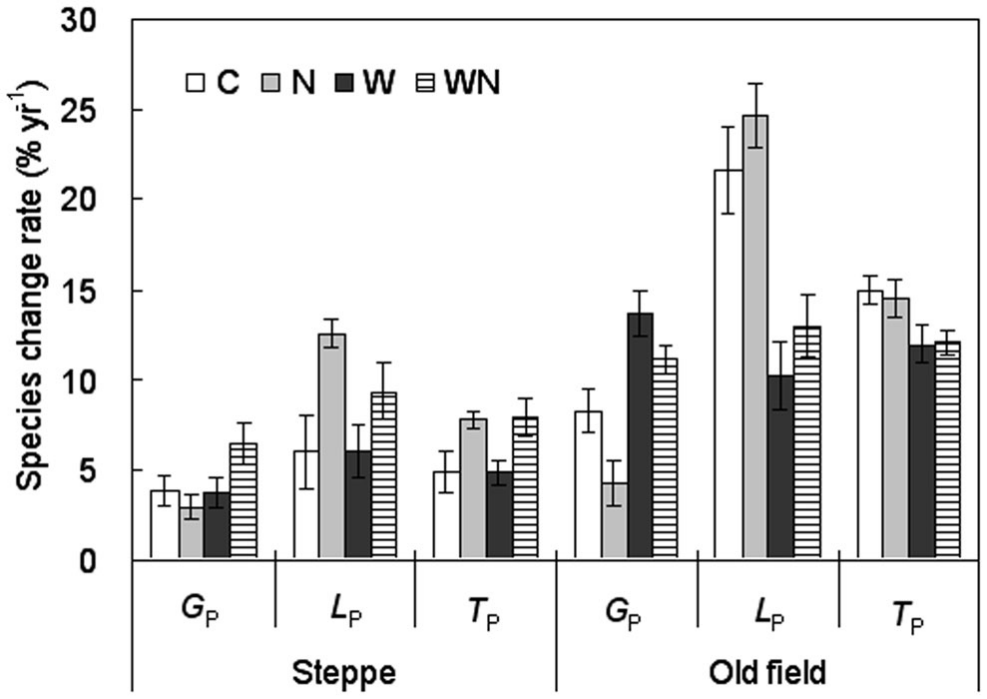
Responses of species gain rate (*G*_p_), loss rate (*L*_p_), and turnover rate (*T*_p_) (mean ± SE) to water and nitrogen addition in a steppe and an old field from 2005 to 2009. See Fig. 1 for treatment abbreviations.

Species changes calculated using data investigated at the end of the experiment (2009) versus data surveyed at the beginning of the experiment (2005) indicated that effects of water and N addition on rates of changes in species differed significantly between the steppe and the old field (Table 3). *G*_p_, *L*_p_ and *T*_p_ in the old field were consistently greater than those in the steppe (*t*-test, all *P<*0.001; Fig. 3). Water addition significantly increased *G*_p_ in the steppe (*P<*0.05) and in the old field (*P<*0.01) but decreased *L*_p_ (*P<*0.001) and *T*_p_ (*P<*0.01) in the old field (Table 3; Fig. 3). N addition significantly enhanced *L*_p_ and *T*_p_ (both *P*<0.05) in the steppe but decreased *G*_p_ (*P*<0.05) in the old field (Table 3; Fig. 3).

**Table 3:** Results (*F*-value) of three-way ANOVAs with a split-plot design on the effects of block (B), water (W) and N addition on species change rate (*G*_p_  =  gain rate; *L*_p_  =  loss rate; *T*_p_  =  turnover rate) in a steppe and in an old field in North China between 2005 and 2009.

Source	Steppe			Old field		
	*G*_p_	*L*_p_	*T*_p_	*G*_p_	*L*_p_	*T*_p_
B	1.82ns	1.29ns	2.61ns	0.47ns	8.02ns	1.90ns
W	10.89*	2.21ns	0.04ns	23.59**	247.14***	16.61**
N	0.94ns	7.78*	9.47*	7.23*	2.74ns	0.04ns
W×N	7.37*	1.60ns	0.01ns	0.38ns	0.01ns	0.10ns

* , **, *** indicate statistically significant difference at *P*<0.05, 0.01, and 0.001, respectively;

ns: non-significant (*P*>0.05).

**Figure 4:**
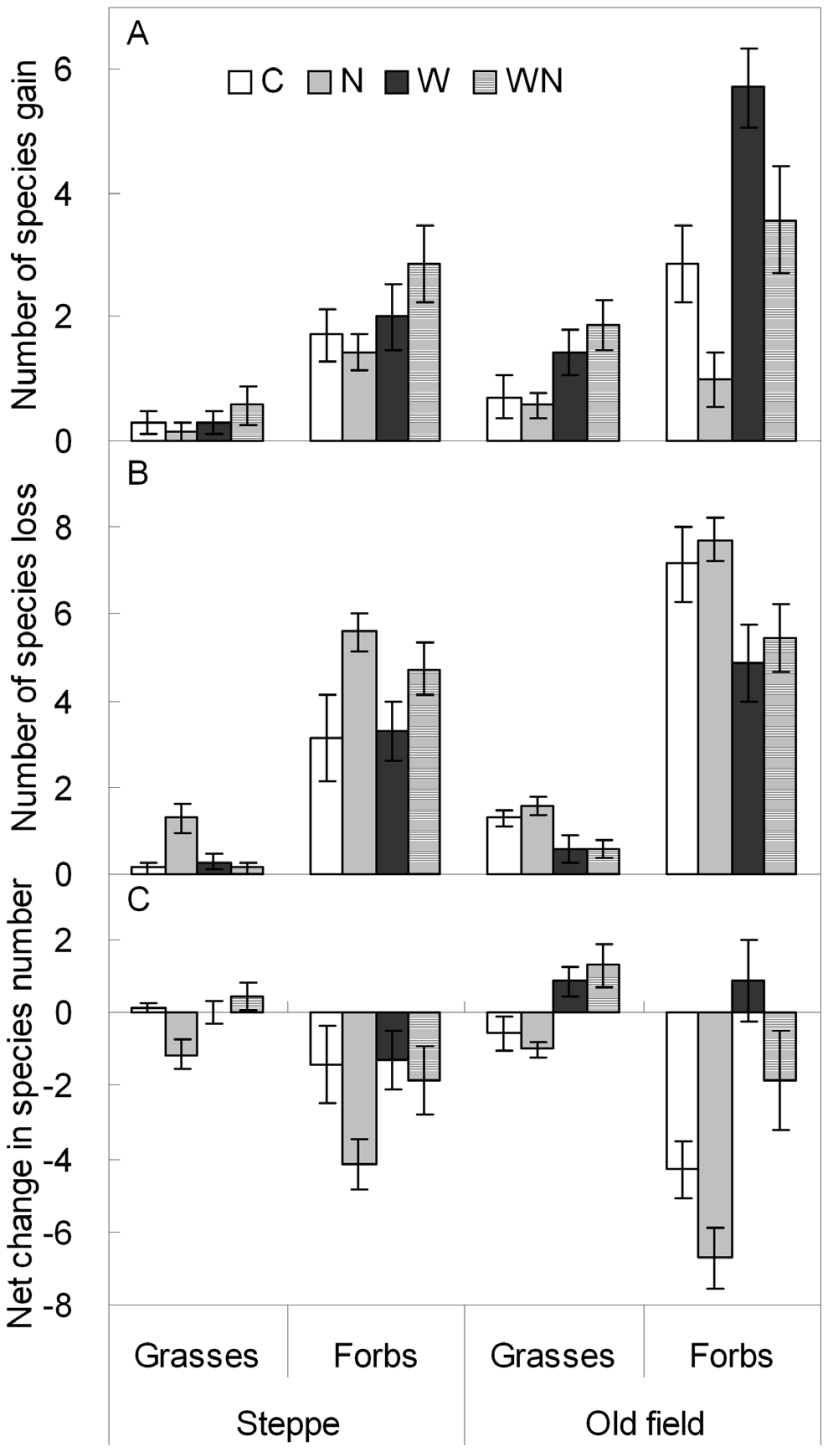
Influences of water and nitrogen addition on numbers of species gain (**A**)**, loss** (**B**)**, and net change** (**C**) **of grasses and forbs (mean ± SE) in a steppe and an old field treated for 5 years.** See Fig. 1 for treatment abbreviations.

Further analyses indicated that responses of species changes at the functional group level to water and N addition differed between the two grassland types (Table 4). Water addition resulted in significant increases in the species number of grasses and forbs gained (both *P*<0.05; Fig. 4A) and, significant decreases in the species number of grasses and forbs lost (*P*<0.01 & 0.05, respectively) (Fig. 4B) (Table 4), and therefore a significant net increase in the species number of grasses (+1.9 species; *P*<0.01) and forbs (+5.0 species; *P*<0.001) in the old field (Fig. 4C). For the steppe, water addition did not affect grasses gain and forbs loss but influenced forbs gain and grasses loss at a *P*<0.1 level (Table 4; Fig. 4A,B). N addition significantly suppressed numbers of forbs gained (*P*<0.05) in the old field, but stimulated numbers of grasses (*P*<0.1) and forbs (*P*<0.05) lost in the steppe (Table 4; Fig. 4A,B). Significant interaction of water and N addition was found only for grasses loss in the steppe (*P*<0.05; Table 4). An overall trend indicated that N addition led to a net decrease in numbers of grasses and forbs in both grassland types (Fig. 4C). However, the combination of water addition and N addition tended to increase the number of grass species and to decrease the number of forb species for both grassland types (Fig. 4C). In both the steppe and the old field, the numbers of species gained, lost and net change for forbs were significant greater than for grasses irrespective of water and N addition treatments (*t*-test, *P*<0.001; Fig. 4).

**Table 4:** Results (*F*-value) of three-way ANOVAs for the effects of block (B), water (W) and N addition on numbers of species gain and loss for grasses and forbs in a steppe and in an old field in North China between 2005 and 2009.

Source	Steppe		Old field		Steppe		Old field	
	Grasses gain	Forbs gain	Grasses gain	Forbs gain	Grasses loss	Forbs loss	Grasses loss	Forbs loss
B	0.46ns	2.43ns	1.68ns	0.60ns	1.36ns	1.38ns	1.62ns	0.99ns
W	0.66ns	4.28Λ	9.48*	11.11*	4.45Λ	0.30ns	16.62**	9.75*
N	0.07ns	0.48ns	0.19ns	6.03*	4.45Λ	8.68*	0.46ns	0.61ns
W×N	0.66ns	1.90ns	0.77ns	0.03ns	7.36*	0.58ns	0.46ns	0.00ns

* , **, *** indicate statistically significant difference at *P*<0.05, 0.01, and 0.001, respectively;

Λ marginally significant difference at *P*<0.1;

ns: *P*>0.1.

### Responses of Functional Group Cover and Litter Accumulation to Water and N Addition

**Figure 5:**
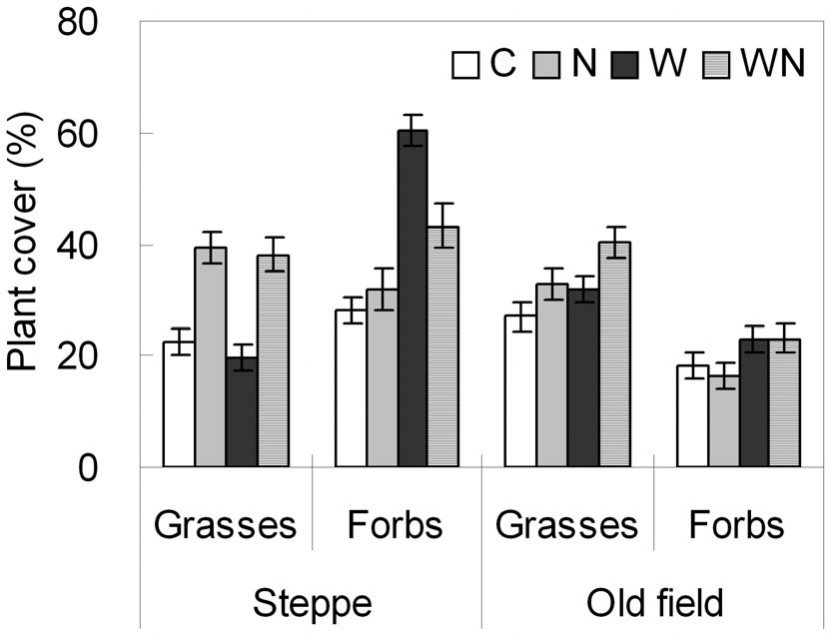
Plant cover (mean ± SE) of grasses and forbs in relation to water and nitrogen addition in a steppe and an old field treated for 5 years. See Fig. 1 for treatment abbreviations.

Water and N additions altered the functional group cover of the community in both the steppe and the old field (Fig. 5). Water addition increased forbs cover by 73.2% (*P*<0.001) in the steppe, and increased both grasses and forbs cover by 20.6% (*P*<0.05) and 33.8% (*P*<0.01) in the old field, respectively (Table 1; Fig. 5). N enrichment enhanced grasses cover by 84.0% in the steppe (*P*<0.01) and by 24.4% (*P*<0.01) in the old field (Table 1; Fig. 5). Linear regression analyses showed that *A. cristatum* (L.) Gaertn. and *S. krylovii* Roshev. accounted for 31.9% and 69.6% of the variations in grasses cover, respectively (both *P*<0.001), and *A. frigida* Willd. for 60.9% of the variations in forbs cover (*P*<0.001) in the steppe. In the old field, the variations in grasses cover were mainly caused by *A. cristatum* (L.) Gaertn. (85.1%; *P*<0.001), while *A. scoparia* Waldst. et Kit. and *Medicago sativa* L. contributed to 21.2% and 35.6% of the variations in forbs cover, respectively (*P*<0.001). N addition increased litter biomass both in the steppe and the old field (*P*<0.05 & 0.1, respectively; Table 1). Water addition markedly promoted litter biomass in the old field (*P*<0.05) but not in the steppe (Table 1).

### Relationships Between Species Change Rate and Community Temporal Stability

**Figure 6:**
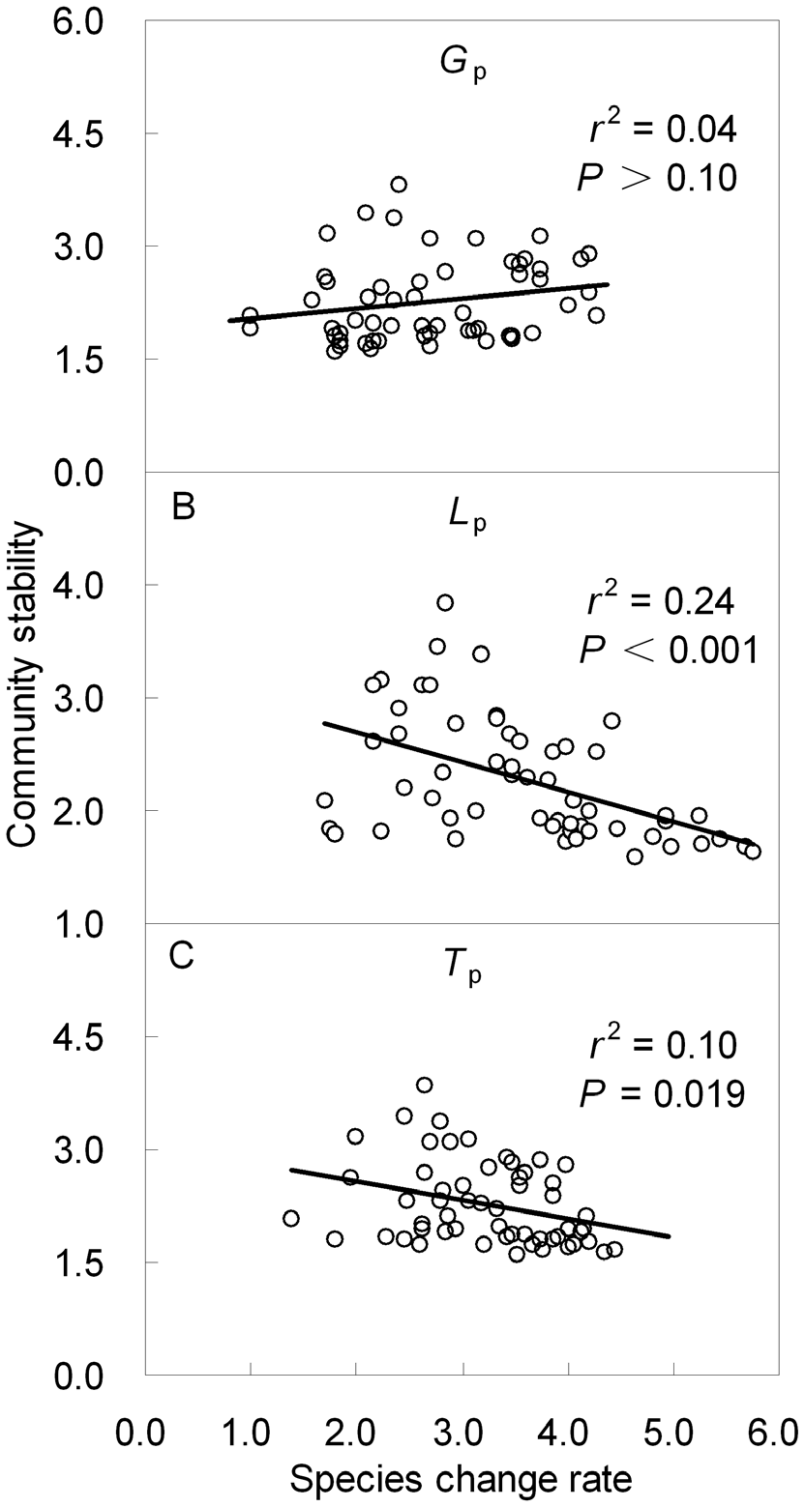
Relationships between temporal stability of community and species gain rate (*G*_p_) (**A**)**, loss rate (** ***L*** **_p_)** (**B**)**, and turnover rate (** ***T*** **_p_)** (**C**)**.** Data were square root transformed, unit of species change rate prior to transformation was % yr^−1^.

Results from linear regression analyses demonstrated that the temporal stability of community was a declining function determined by both species loss rate (*r* ^2^ = 0.24, *P<*0.001; Fig. 6B) and species turnover rate (*r* ^2^ = 0.10, *P  = *0.019; Fig. 6C). The relationship between species gain rate and temporal stability was not significant (*P*>0.10; Fig. 6A).

## Discussion

During the 5-year study period, both the steppe and the old field suffered from diversity loss with marked interannual variations (Fig. 1; Table 1). The interannual variations of species richness (Fig. S1A) might have been resulted from the substantial variations in the amount of precipitation during the growing season in these semi-arid ecosystems. Precipitation from May to July varied from 338.3 mm in 2006 to 78.0 mm in 2007, with a mean value of 176.0 mm for the study period of five years. Tilman [36] proposed that the durative climatic change may modify both plant life history and competitive abilities of species, leading to loss of equilibrium of community composition and change in species richness, and alteration of plant succession trajectory.

The increase in species richness in water addition plots in our study is similar to previous studies carried out in a steppe in the same region [11], in an annual grassland in California [7] and in a secondary grassland in Kansas in the USA [27]. However, there are greater differences in natural precipitation and/or grassland types between our experiment and the other three studies. N addition had no effects on species richness in the present study, which was inconsistent with majority of previous studies but in line with the findings gained from four artificial communities in the UK [37]. The absence of statistical significance of N addition on species richness (Table 1; Fig. 1) may be partly resulted from the relatively short study period of 5 years. Species richness was stimulated by N addition during the first two years, and then decreased for the following years (data not shown). The effect of N addition on species richness was not significant when analyzing the pooled data across the 5-year treatment period (Table 1). These observations suggest that the response of species richness to N addition in semi-arid grasslands may require relatively long-term studies, but the effects of water addition on species richness occurred much more rapidly. Alternatively, the effects of N on species richness are also likely dependent upon soil water conditions and species composition. The lower species richness in the old field than in the steppe is probably because the more competitive exclusion of *A. cristatum* to other species and the shortage of diverse propagules in the old field.

Changes in environmental conditions may cause changes in species turnover [2], [38]. Using natural experiments without water manipulation, Anderson [3] did not investigate the relative contributions of species gain and loss to changes in species richness but found that there was a close relationship between species turnover and dry season rainfall in grasslands. In our study sites, water addition affected the rate of interannual species gain, loss and turnover in both the steppe and the old field. However, there were significant differences among year-to-year dynamics and no consistent pattern was found (Table 2; Fig. 2). The short-term dynamics may be controlled, in great degree, by environmental fluctuations, especially precipitation. Relative long-term observation may provide more reliable information for the impacts of treatments. Water addition alleviates the limitation of soil moisture on species, leading to coexistence of more species (Table 1; Fig. 1, S1B) and reduction in species loss (Table 4; Fig. 4B). Hence, the *G*_p_ increased and the *L*_p_ decreased in the water addition plots (Table 3; Fig. 3).

Our study showed that N addition enhanced grasses cover but reduced forbs cover (Table 1; Fig. 5). Most of the grasses are taller than forbs in these grasslands, and therefore, can produce greater living biomass (Xu ZW, unpublished data) and litter biomass compared to forbs. The increased litter biomass in N addition plots may limit the establishment of new species [7], [39], resulting in decreased *G*_p_ and increased *L*_p_ (Table 1,3; Fig. 3). In line with our results, previous studies found only few new species gained but more existed species lost under N enrichment in a herb-rich woodland in Australia [14] and in four grasslands in Minnesota [40]. Indeed, previous studies have already documented that N enrichment reduced species richness due to the suppressive effects of increased litter biomass on the seedling establishment [39], [41]. The greater species changes in forbs than in grasses (*t*-test; Fig. 4), irrespective of grassland types and treatments, indicated that the species richness and composition in the semi-arid grasslands are mainly determined by the response of forbs.

Overall, either water or N can act independently as driver of plant community dynamics, and water condition also inﬂuenced community sensitivity to N, and *vice versa* [42], [43]. The significant interactions between water and N on species richness, forbs cover, and species turnover indicated that the effects of N on plant community are strongly mediated by water availability in the temperate grasslands (Table 4; Fig. 4B). This may be explained by (1) N transformation controlled directly by soil water conditions [44], and (2) reduced water availability caused by increased growth rate of plants under N addition [7], [45]. These findings suggest that predicting the responses of grasslands to global change drivers should take into account the interactions among environmental factors.

The present study showed that the species richness of annuals and biennials declined from 2005 to 2009 in both the steppe and the old field (Fig. S2). This finding indicated that the replacement of short-lived species by perennials is a common trend in natural ecosystems over time. During the succession of plant communities, pioneer species will be replaced by later successional species with longer life cycles [46]. Rate in replacement of plant species is higher during the early successional stages than the late stages [2], [47], which is supported by our results that species change rate in the old field was greater than in the steppe since plant community in the abandoned old field was at an earlier stage of succession than that in the steppe. It is expected that life history strategies and traits of plants will shift from *r*-strategy colonizers to competitively superior *k*-strategists during succession series [48]–[50]. In line with this expectation, the present study found that the majority of the plant species in the steppe were the *k*-strategy colonizers (i.e. perennial species) with high efficiency for habitat exploitation. These species are relatively stable and can sustain for long term under fluctuating environmental conditions. But in the old field, there were relatively more annuals and biennials which are mainly opportunists and *r*-strategists with high dispersal abilities and high stress tolerance. The different strategies in life history also partly resulted in divergence in rate of species change between the two grassland types (Fig. 3, S2). Differences in responses of plant functional groups and differences in rates of species change between the steppe and the old field suggest that the grassland ecosystems with different land use history in northern China may develop with different processes or trajectories under future environmental changes.

The present study found negative relationships between temporal stability of community and rate of species loss and/or turnover (Fig. 6), which is supported by findings of Hillebrand *et al*. [51] that the temporal stability of biomass production significantly decreased when community species composition showed higher temporal turnover. Anderson [2] and McIntyre and Lavore [52] suggested that community stability increased as rates of species change decreased with time. Our results also indicated that water addition increased the temporally compositional stability, while N addition decreased the stability.

The present results support our initial hypothesis and provide direct experimental evidence for opposite effects of water and N addition on species turnover rate in temperate semi-arid grasslands in northern China. Both increased rate of species gain and decreased rate of species loss contributed to the enhanced species richness when water availability was improved. In contrast, N addition caused a decrease in the rate of species gain and an increase in the rate of species loss. However, effects of water and N availabilities on plant diversity and species turnover also depend on grassland types and/or land-use history. Our results demonstrated the relative contributions of species gain and loss to the dynamic change of species richness in semi-arid grasslands under future climate change, and highlighted the complexity of the ecological consequences of concurrent increases in precipitation and N deposition in the temperate grasslands in North China.

## Supporting Information

Figure S1 A, Interannual variations of mean species richness across treatments; B, Treatment effects on mean species richness across the study period of 5 years. C: control, N: nitrogen addition, W: water addition, WN: combination of water and nitrogen addition.
(TIF)

Figure S2 Species richness of annuals and biennials (AB) and perennials (PE) from 2005 to 2009 in a steppe and an old field. C: control, N: nitrogen addition, W: water addition, WN: combination of water and nitrogen addition.
(TIF)
