# Supplementary figures and images for: Effects of Water and Nitrogen Addition on Species Turnover in Temperate Grasslands in Northern China
Source: PLoS One. 2012 Jun 29;7(6):e39762. doi: 10.1371/journal.pone.0039762 (PMC3387244; doi:10.1371/journal.pone.0039762)

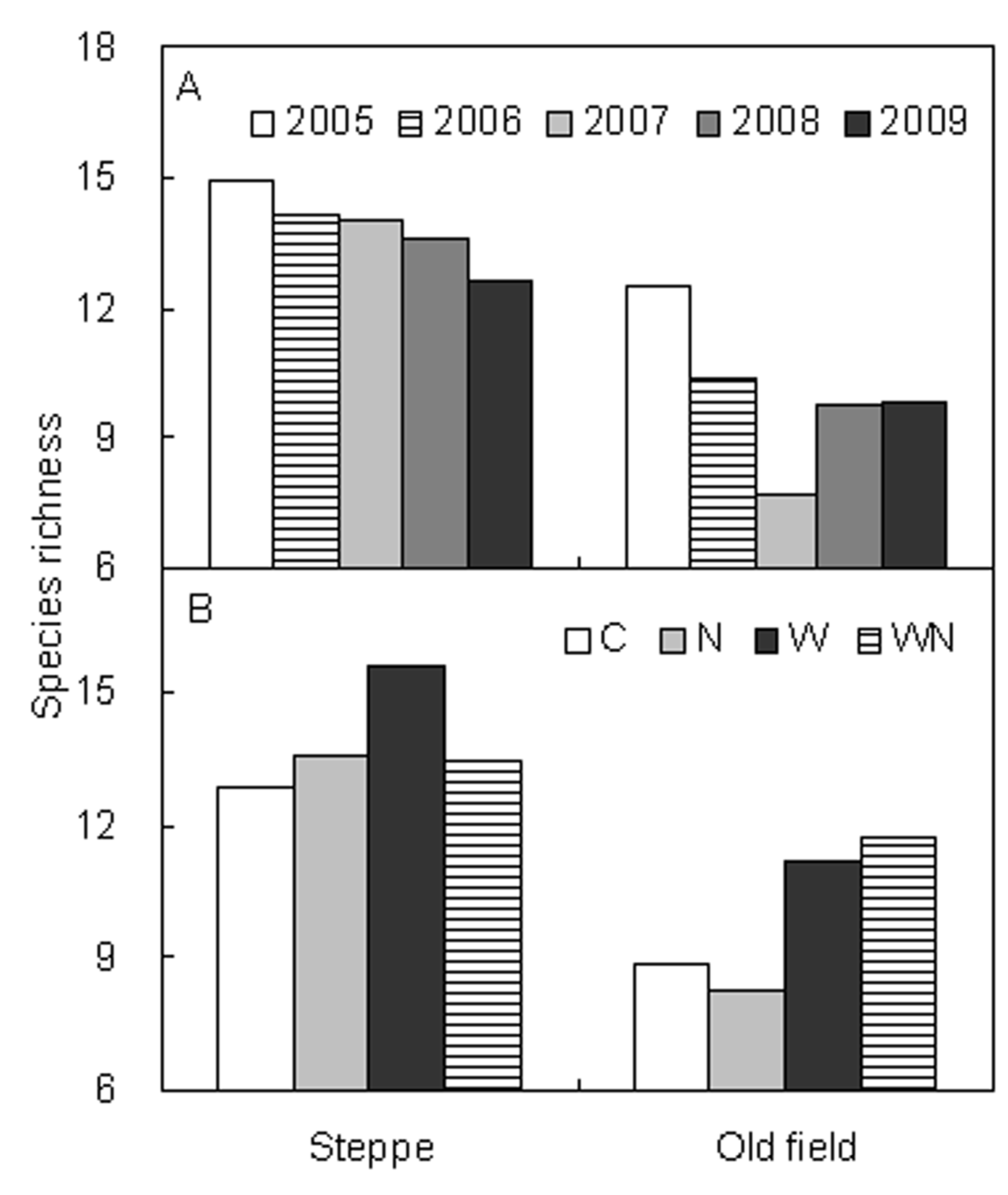

Supplement: Figure S1 — A, Interannual variations of mean species richness across treatments; B, Treatment effects on mean species richness across the study period of 5 years. C: control, N: nitrogen addition, W: water addition, WN: combination of water and nitrogen addition. (TIF) [file pone.0039762.s001.tif]

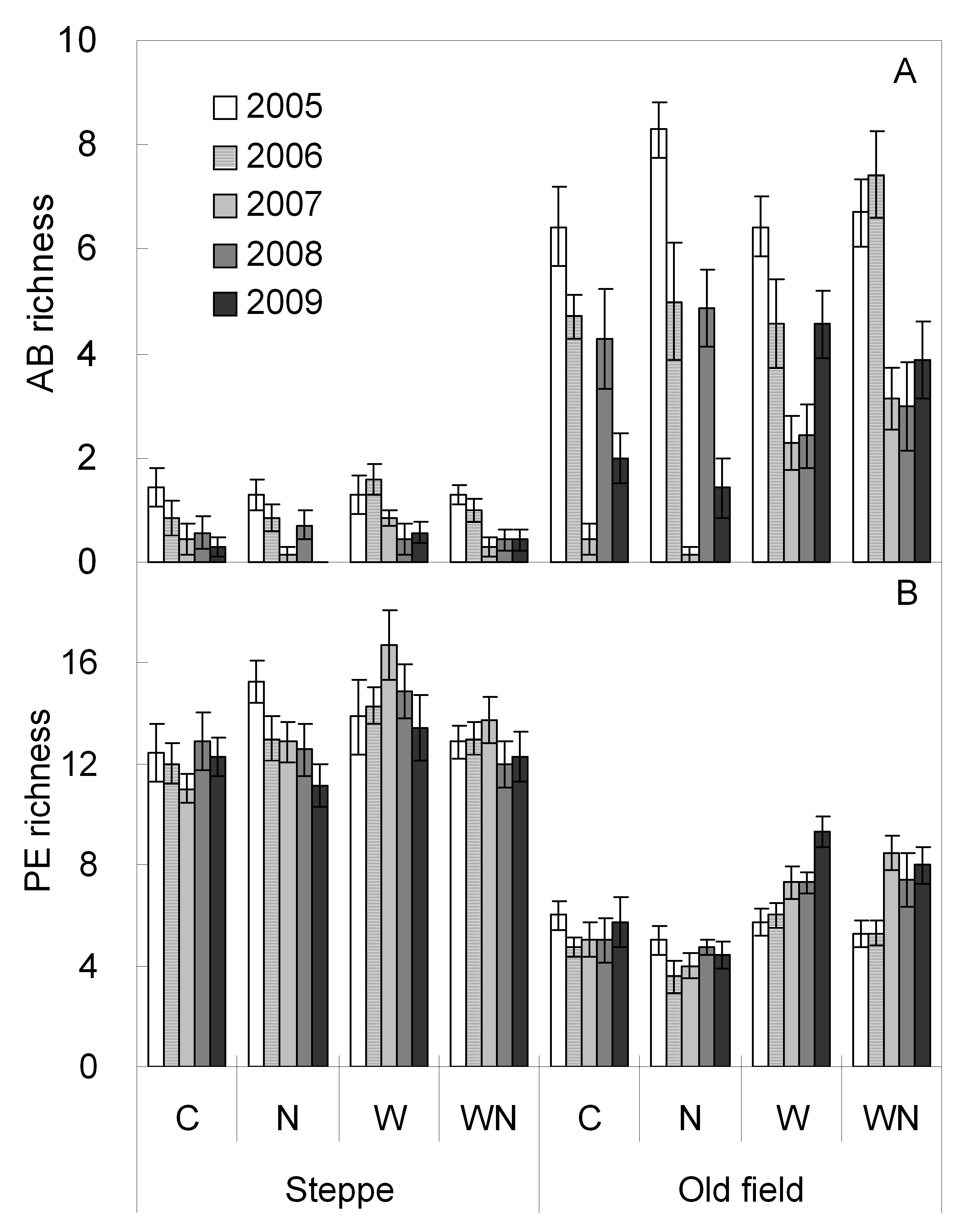

Supplement: Figure S2 — Species richness of annuals and biennials (AB) and perennials (PE) from 2005 to 2009 in a steppe and an old field. C: control, N: nitrogen addition, W: water addition, WN: combination of water and nitrogen addition. (TIF) [file pone.0039762.s002.tif]
